# Imipenem/Relebactam Plus Aztreonam: First Reported Use in MDR *Klebsiella pneumoniae* Sternal Infection Complicated by Bacteremia

**DOI:** 10.3390/antibiotics14101007

**Published:** 2025-10-10

**Authors:** Luca Pipitò, Raffaella Rubino, Rita Immordino, Eleonora Bono, Teresa Fasciana, Celestino Bonura, Giovanni Maurizio Giammanco, Vincenzo Argano, Antonio Cascio

**Affiliations:** 1 Department of Health Promotion, Mother and Child Care, Internal Medicine and Medical Specialties “G D’Alessandro”, University of Palermo, 90133 Palermo, Italy; eleonora.bono@community.unipa.it (E.B.); teresa.fasciana@unipa.it (T.F.); celestino.bonura@unipa.it (C.B.); giovanni.giammanco@unipa.it (G.M.G.); 2 Infectious and Tropical Disease Unit and Sicilian Regional Reference Center for the Fight Against AIDS, AOU Policlinico “P. Giaccone”, 90133 Palermo, Italy; raffaella.rubino@policlinico.pa.it; 3 Antimicrobial Stewardship Team, AOU Policlinico “P. Giaccone”, 90127 Palermo, Italy; 4 Microbiology and Virology Unit, AOU Policlinico “P. Giaccone”, 90133 Palermo, Italy; rita.immordino@policlinico.pa.it; 5 Unit of Cardiac Surgery, University of Palermo, 90127 Palermo, Italy; vincenzo.argano@policlinico.pa.it

**Keywords:** carbapenem-resistant *Klebsiella pneumoniae*, bacteremia, imipenem/cilastatin/relebactam, ceftazidime-avibactam resistance, aztreonam, KPC (*Klebsiella pneumoniae* carbapenemase), metallo-β-lactamases

## Abstract

Background: Carbapenem-resistant *Klebsiella pneumoniae* (CRKP) poses a significant therapeutic challenge, particularly when multiple resistance mechanisms, such as metallo-β-lactamases (MBLs) and *Klebsiella pneumoniae* carbapenemase (KPC), coexist. Case description: We describe a case of a 51-year-old male with a post-sternotomy surgical site infection and concurrent bacteremia caused by a CRKP. Sternal swab and mediastinal liquid culture results highlighted CRKP harboring *bla*_NDM_ and *bla*_KPC_ genes, while the blood isolate showed *bla*_CTX_ and *bla*_KPC_, indicating phenotypic resistance to ceftazidime-avibactam. All the strains exhibited phenotypic susceptibility to meropenem-vaborbactam (MEV), despite having a high minimum inhibitory concentration. Following clinical failure of MEV-based therapy, combination treatment with aztreonam (ATM) and imipenem/cilastatin/relebactam (IMI/REL), plus gentamicin, was initiated. Therapy was well tolerated and resulted in microbiological eradication and full clinical recovery. The patient completed 49 days of ATM and IMI/REL without relapse over a 3-month follow-up period. This is, to the best of our knowledge, the first reported case of IMI/REL being used in combination with ATM.

## 1. Introduction

Carbapenem-resistant *Klebsiella pneumoniae* (CRKP) infections and sepsis represent a significant public health challenge due to their high associated mortality and limited therapeutic options [1,2,3].

The World Health Organization has classified CRKP within the “critical priority” group of pathogens in its Bacterial Priority Pathogen List, with its ranking rising from fifth position in 2017 to first in 2024 [4].

The primary mechanism of resistance in CRKP involves the production of β-lactamase enzymes, as *Klebsiella pneumoniae* carbapenemase (KPC)-type class A β-lactamases and class B metallo-β-lactamases (MBLs) [5].

Therapeutic strategies for infections caused by KPC-producing *K. pneumoniae* include the use of β-lactam/β-lactamase inhibitor combinations such as ceftazidime-avibactam (CAZ-AVI), meropenem-vaborbactam (MEV), and imipenem/cilastatin/relebactam (IMI/REL). In contrast, for MBL-producing strains, the combination of aztreonam (ATM) with CAZ-AVI is currently considered the preferred treatment option. Alternative agents include cefiderocol and eravacycline [6].

ATM is intrinsically stable against hydrolysis by MBLs but remains susceptible to degradation by other β-lactamases. When combined with avibactam, ATM is protected from these additional enzymatic activities [7].

However, the co-production of multiple β-lactamases, including both MBLs and avibactam-resistant KPC variants, can compromise the efficacy of this regimen. KPC variants (e.g., KPC-31, -33, -44, -50, -86) are known to confer resistance to avibactam [8].

In cases of complex resistance profiles where cefiderocol is not employed, alternative combination therapies, such as ATM combined with either relebactam or vaborbactam, may be considered as rescue options. These agents may potentially protect ATM from hydrolysis by avibactam-resistant KPC enzymes [9]. Furthermore, the addition of imipenem to ATM and CAZ-AVI was highly synergistic in vitro in the case of long filamentous cell formation induced by PBP3 inhibition [10,11].

Despite this theoretical rationale, clinical data on the use of ATM in combination with IMI/REL for treating multidrug-resistant (MDR) *K. pneumoniae* remain absent in the literature.

Herein, we describe a case of successful treatment with IMI/REL and ATM in a patient with a sternal surgical site infection and concurrent bacteremia caused by carbapenem-resistant *Klebsiella pneumoniae* (CRKP) strains, both phenotypically resistant to CAZ-AVI. Molecular testing using BioFire^®^ (bioMérieux, Salt Lake City, Utah, USA) identified both *bla*_NDM_ and *bla*_KPC_ genes in the sternal surgical isolate, and *bla*_KPC_ and *bla*_CTX_ in the blood isolate. To the best of our knowledge, this is the first reported case of IMI/REL being combined with ATM for the treatment of multidrug-resistant *K. pneumoniae* infections.

## 2. Case Report

### 2.1. Patient History

A 51-year-old man was admitted with sternal wound dehiscence following surgical repair of a Stanford type A aortic dissection. His medical history included obesity, obstructive sleep apnea syndrome, and hypertension. Chest computed tomography (CT) revealed a retrosternal fluid collection. The physical examination revealed separation of the surgical margins, sternal instability, and signs of local inflammation. Laboratory tests indicated a markedly elevated C-reactive protein (CRP) level of 276 mg/L (normal < 5 mg/L). Vital signs were as follows: temperature 36 °C, blood pressure 115/67 mmHg, pulse rate 94 bpm, oxygen saturation 99% on room air, and respiratory rate 24 breaths/min. Empiric antibiotic therapy with vancomycin (1 g twice daily) and ceftriaxone (2 g daily) was initiated. The patient underwent sternal resynthesis followed by vacuum-assisted closure (VAC) therapy. Intraoperatively, 400 mL of serosanguineous fluid was drained, and all dislodged steel wires were removed. Complete sternal diastasis was confirmed. The sternal edges were debrided and reconstructed using the Robicsek technique, and a pericardial drain was placed. New interrupted steel wires were inserted for sternal fixation. The patient was admitted to the intensive care unit (ICU) for five days postoperatively.

### 2.2. Microbiological Findings and Treatment

During the patient’s ICU stay, a fever developed. Bronchoalveolar lavage (BAL) cultures grew CRKP, which was resistant to all tested antibiotics except gentamicin and MEV, and detected the *bla*_NDM_ and *bla*_KPC_ genes by polymerase chain reaction (PCR), as shown in Table 1. Given the downward trend in CRP from a peak of 374 mg/L and the absence of other signs of sepsis, targeted treatment was initially deferred. Subsequently, CRKP was also isolated from deep sternal wound swabs and mediastinal fluid (Table 1). Molecular analysis by PCR confirmed the presence of both *bla*_NDM_ and *bla*_KPC_ genes in all isolates.

Due to persistent fever, extended-infusion MEV and fosfomycin were initiated. However, after six days, there was no clinical improvement, and CRKP was again isolated from deep sternal tissue and blood cultures. Molecular testing by PCR revealed the presence of *bla*_NDM_ in the former and both *bla*_KPC_ and *bla*_CTX-M_ genes in the latter. Given the isolation of a CAZ-AVI-resistant *KPC* variant from blood cultures, along with the detection of co-harboring *bla*_NDM_ and *bla*_KPC_ genes in the other samples, combination therapy with ATM (2 g every 6 h), IMI/REL (1.25 g every 6 h), and gentamicin (3 mg/kg once daily) was initiated. ATM and IMI/REL were continued for 7 weeks, while gentamicin was discontinued after 3 weeks.

### 2.3. Outcome

After 14 days of therapy, both blood cultures and deep wound swabs were negative. The patient showed steady clinical improvement and remained afebrile. At the end of antibiotic therapy, he was discharged home in stable condition, continuing VAC therapy. A follow-up thoracic CT scan showed resolution of the infection, and no recurrence was observed during a 3-month follow-up period.

## 3. Discussion

The treatment of CRKP infections represents a major clinical challenge, particularly when multiple resistance mechanisms coexist. In our case, we observed several CRKP isolates from different anatomical sites, all exhibiting nearly identical phenotypic resistance profiles, with the exception of a MIC for MEV in the isolate from BAL and the susceptibility to an increased dose of meropenem in the isolate from blood and sternal biopsy. Notably, there was a paradoxical discordance between molecular and phenotypic findings. While molecular testing consistently detected both *bla*_NDM_ and *bla*_KPC_ genes (*bla*_NDM_ was absent in the blood isolate, and *bla*_KPC_ was absent in the sternal biopsy isolate), all isolates remained phenotypically susceptible to MEV, although with a high MIC (8/8) according to the European Committee on Antimicrobial Susceptibility Testing breakpoints. This discrepancy may be explained by inducible or non-functional *bla*_NDM_ expression [12,13,14,15]. For example, the insertion of IS10 into *bla*_NDM-1_ has been associated with a lack of expression [12] or with the production of a non-functional NDM variant [13], leading to a mismatch between genotypic and phenotypic findings in *K. pneumoniae*. Qin et al. reported a case of recurrent *K. pneumoniae* pneumonia in which a deletion within the upstream promoter region of *bla*_NDM-1_ impaired gene expression, resulting in phenotypic susceptibility to carbapenems. However, in vitro bactericidal assays and a murine infection model demonstrated that *K. pneumoniae* strains harboring silent *bla*_NDM-1_ exhibited significant tolerance to carbapenem-mediated killing [14]. Further “in vitro” studies have shown that Gram-negative pathogens can upregulate *bla*_NDM-1_ expression over time under carbapenem pressure [15,16].

Thus, exposure to agents such as MEV could induce *bla*_NDM_ expression, potentially leading to treatment failure despite initial susceptibility. Given the patient’s clinical deterioration and MEV failure, as well as the possibility of inducible NDM activity, we initiated combination therapy with IMI/REL and ATM.

CZA plus ATM is considered first-line therapy for MBL-producing *Enterobacterales*, with both in vitro and clinical studies confirming its safety and efficacy [6,17,18]. However, in our case, this option was not pursued because of the isolation of a bloodstream *K. pneumoniae* strain harboring a CZA-resistant KPC variant, which remained phenotypically susceptible only to high-dose meropenem and MEV. Furthermore, isolates from the sternal swab and mediastinal fluid co-harbored *bla*_NDM_ and *bla*_KPC_, and it remained unclear whether this *bla*_KPC_ was resistant to avibactam.

One well-described mechanism of resistance to CZA among KPC-producing strains involves structural alterations in the Ω-loop of the β-lactamase. This region is crucial for maintaining the active-site architecture through a network of hydrogen bonds that stabilize key residues and facilitate the binding of avibactam. Mutations affecting the Ω-loop, particularly substitutions at position 179 (e.g., D179N), destabilize these interactions and increase local flexibility. As a result, the inhibitory effect of avibactam is reduced. Importantly, these mutations also decrease the enzyme’s catalytic activity against carbapenems [19] and do not impair the binding of vaborbactam or relebactam [19]. Nonetheless, MEV was not considered in combination with ATM for our patient, as the earlier regimen of MEV plus fosfomycin had failed, and bacteremia due to *K. pneumoniae* had developed.

In our case, the rationale behind combining ATM and IMI/REL was based on the complementary spectrum of action of the two drugs. ATM is a monobactam intrinsically stable against hydrolysis by MBLs, such as NDM, VIM, and IMP, because its structure does not fit efficiently into the zinc-dependent active site of these enzymes [7]. However, ATM is readily hydrolyzed by serine β-lactamases (SBLs), including KPC, extended-spectrum β-lactamases (ESBLs), and AmpC, which are frequently co-produced by MBL-producing Enterobacterales. In this context, relebactam acts as a potent inhibitor of class A and class C β-lactamases, effectively blocking KPC and AmpC activity and thereby preventing the degradation of ATM [7]. Since MBLs cannot inactivate ATM, and relebactam neutralizes SBLs, the combination restores ATM activity, allowing it to bind penicillin-binding proteins (particularly PBP-3) and exert bactericidal activity on the bacterial cell wall. Importantly, relebactam has also shown activity against CZA-resistant KPC variants [7,20,21]. In our case, relebactam therefore served a dual function: protecting ATM from SBL-mediated hydrolysis and maintaining efficacy in the presence of KPC variants that confer resistance to CZA [19].

In vitro studies support the synergistic potential of ATM combined with relebactam [21,22]. Fu et al. reported strong synergy of ATM in combination with CZA, MEV, and IMI/REL against 12 MBL- and/or KPC-producing Enterobacterales, including nine *K. pneumoniae* strains [21]. In a larger cohort of carbapenemase-producing Gram-negative bacteria, ATM MICs were reduced when combined with relebactam in isolates co-producing ESBL or AmpC enzymes, as well as MBLs such as IMP, NDM, GES, or OXA-48 [22]. Nevertheless, the use of IMI/REL plus ATM is not yet established in clinical practice, and to our knowledge, no previous reports of this combination have been published.

In our patient, this regimen was administered for 49 days without any adverse events, resulting in both microbiological eradication and sustained clinical recovery. Gentamicin was added during the first three weeks to provide additional synergy with β-lactams in the treatment of Gram-negative infections [23]. Cefiderocol and eravacycline were also considered as alternative therapies; however, they were not used due to the unavailability of susceptibility testing at the time and the favorable response obtained with ATM plus IMI/REL [6]. This favorable outcome highlights the potential of ATM–IMI/REL as a salvage therapy for MDR infections caused by Enterobacterales co-producing MBLs and avibactam-resistant KPC variants.

## 4. Conclusions

This case highlights the complexity of managing infections caused by *K. pneumoniae*, which co-produces multiple resistance determinants, including MBLs and avibactam-resistant KPC variants, and underscores the clinical significance of discrepancies between molecular and phenotypic testing. The successful use of ATM plus IMI/REL in the absence of established guidelines suggests that this combination may represent a viable salvage option in critically ill patients with MDR infections involving complex resistance mechanisms. It may be particularly valuable in cases where Enterobacterales produce both MBLs and CZA-resistant KPC variants, or when multiple isolates with different resistance profiles are present, as was the case in our study. Further clinical studies are needed to better define the role and effectiveness of this therapeutic approach in high-risk scenarios.

## Figures and Tables

**Table 1:** Antibiogram interpretation of *K. pneumoniae* isolates was performed according to European Committee on Antimicrobial Susceptibility Testing breakpoints. Susceptibility data for cefiderocol, IMI/REL, ATM/AVI, and eravacycline were not routinely tested and were unavailable at the time of the clinical case.

	BAL (KPC + NDM) 24 March		Swab (KPC + NDM) 1 April		Liquid (KPC + NDM) 1 April		Biopsy (NDM) 8 April		Blood (KPC + CTX-M) 11 April	
Drug	MIC	Susc	MIC	Susc	MIC	Susc	MIC	Susc	MIC	Susc
Ak	>16	R	>16	R	>16	R	>16	R	>16	R
ATM	>16	R	>16	R	>16	R	>16	R	>16	R
FEP	>8	R	>8	R	>8	R	>8	R	>8	R
CAZ	>16	R	>16	R	>16	R	>16	R	>16	R
CAZ-AVI	>8/4	R	>8/4	R	>8/4	R	>8/4	R	>8/4	R
C/T	>4/4	R	>4/4	R	>4/4	R	>4/4	R	>4/4	R
CRO	>4	R	>4	R	>4	R	>4	R	>4	R
CIP	>1	R	>1	R	>1	R	>1	R	>1	R
CST			≤0.5	S	≤0.5	S	≤0.5	S	≤0.5	S
GEN	≤1	S	≤1	S	≤1	S	≤1	S	≤1	S
IMI	>8	R	>8	R	>8	R	>8	R	>8	R
LFX	>1	R	>1	R	>1	R	>1	R	>1	R
MER	>16	R	>16	R	>16	R	8	I	8	I
MEV	4/8	S	8/8	S	8/8	S	8/8	S	8/8	S
TZP	>64/4	R	>64/4	R	>64/4	R	>64/4	R	>64/4	R
TMP-SMX	>4/76	R	>4/76	R	>4/76	R	>4/76	R	>4/76	R

BAL, bronchoalveolar lavage; MIC, minimal inhibitory concentration; KPC, *Klebsiella pneumoniae* carbapenemase; NDM, New Delhi metallo-beta-lactamase; CTX-M, Cefotaximase-Munich; Susc, susceptibility; Ak, amikacin; ATM, aztreonam; FEP, cefepime; CAZ, ceftazidime; CAZ-AVI, ceftazidime-avibactam; C/T, ceftolozane tazobactam; CRO, ceftriaxone; CIP, ciprofloxacin; CST, colistin; GEN, gentamicin; IMI, imipenem; LFX, levofloxacin; MER, meropenem; MEV, meropenem-vaborbactam; TZP, piperacillin-tazobactam; TMP-SMX, trimethoprim-sulfamethoxazole; S, susceptible; R, resistant; I, susceptible increase dose.

## Data Availability

Data will be made available upon request.

## Abbreviations

The following abbreviations are used in this manuscript:

ATM	Aztreonam
BAL	Bronchoalveolar lavage
CAZ-AVI	Ceftazidime avibactam
CRKP	Carbapenem resistant *Klebsiella pneumoniae*
CRP	C-reactive protein
CT	Computed tomography
ICU	Intensive care unit
IMI/REL	Imipenem relebactam
KPC	*Klebsiella pneumoniae* carbapenemase
MBL	Metallo-β-lactamases
MDR	Multidrug resistant
MEV	Meropenem vaborbactam
MIC	Minimum inhibitory concentration
NDM	New Delhi metallo-beta-lactamase
VAC	vacuum-assisted closure
